# Deciphering the Intracellular Fate of *Propionibacterium acnes* in Macrophages

**DOI:** 10.1155/2013/603046

**Published:** 2013-06-05

**Authors:** Natalie Fischer, Tim N. Mak, Debika Biswal Shinohara, Karen S. Sfanos, Thomas F. Meyer, Holger Brüggemann

**Affiliations:** ^1^Unit Molecular Microbial Pathogenesis, Pasteur Institute, 75724 Paris, France; ^2^Department of Molecular Biology, Max Planck Institute for Infection Biology, 10117 Berlin, Germany; ^3^Department of Biomedicine, Aarhus University, Wilhelm Meyers Allé 4, Bartholin Building, 8000 Aarhus C, Denmark; ^4^Department of Environmental Health Sciences, Bloomberg School of Public Health, Johns Hopkins University, Baltimore, MD 21231, USA; ^5^Department of Pathology, Johns Hopkins University School of Medicine, Baltimore, MD 21231, USA

## Abstract

*Propionibacterium acnes* is a Gram-positive bacterium that colonizes various niches of the human body, particularly the sebaceous follicles of the skin. Over the last years a role of this common skin bacterium as an opportunistic pathogen has been explored. Persistence of *P. acnes* in host tissue has been associated with chronic inflammation and disease development, for example, in prostate pathologies. This study investigated the intracellular fate of *P. acnes* in macrophages after phagocytosis. In a mouse model of *P. acnes*-induced chronic prostatic inflammation, the bacterium could be detected in prostate-infiltrating macrophages at 2 weeks postinfection. Further studies performed in the human macrophage cell line THP-1 revealed intracellular survival and persistence of *P. acnes* but no intracellular replication or escape from the host cell. Confocal analyses of phagosome acidification and maturation were performed. Acidification of *P. acnes*-containing phagosomes was observed at 6 h postinfection but then lost again, indicative of cytosolic escape of *P. acnes* or intraphagosomal pH neutralization. No colocalization with the lysosomal markers LAMP1 and cathepsin D was observed, implying that the *P. acnes*-containing phagosome does not fuse with lysosomes. Our findings give first insights into the intracellular fate of *P. acnes*; its persistency is likely to be important for the development of *P. acnes*-associated inflammatory diseases.

## 1. Introduction

The Gram-positive bacterium *Propionibacterium acnes* (*P. acnes*) is ubiquitously present in various niches of the human body, such as the oral cavity, the gastrointestinal tract, and the conjunctiva. It is most prevalent on the human skin, where it colonizes sebaceous follicles of the face and back [1]. Due to its intimate association with sebaceous follicles, it has been studied as the possible etiological agent of the skin disorder acne vulgaris, but clear-cut proof for *P. acnes*' involvement is still scarce [2–7]. 

Over the last few years *P. acnes*' role as an opportunistic pathogen has become more prominent. The bacterium is associated with several inflammatory diseases, such as endophthalmitis, endocarditis, osteomyelitis, sarcoidosis, keratitis, and the SAPHO syndrome and inflammatory conditions linked to surgery or the implantation of foreign devices, such as prosthetic aortic valves, prosthetic hip and shoulder implants [8]. *P. acnes* is also suspected to cause chronic inflammation in the human prostate that might be associated with the development of prostate cancer. Several studies have reported the detection and isolation of *P. acnes* from prostate tissue samples of diseased patients [9–13]. *In vitro* experiments showed that *P. acnes* can invade the epithelial cell lines A549, HEK293T [14] and the prostate epithelial cell line RWPE1 [9, 15]. 

Intracellular detection of *P. acnes* and evidence for inflammation in *P. acnes*-infected tissues suggest that the intracellular presence of *P. acnes* supports its long-term persistency in the host, which could result in a chronic inflammatory state. However, persistency is not easily achieved as the host has powerful means to eradicate microbial intruders. An important line of defence is the recruitment of specialized phagocytic cells, such as macrophages and neutrophils. These cells engulf microorganisms into phagosomes, specialized compartments, which undergo concurrent interactions and exchanges with compartments of the endocytic pathway. Thereby the phagosome gradually becomes a highly acidic and oxidizing environment, enriched in hydrolytic enzymes, which finally enables degradation of the microorganism [16–18]. Intracellular pathogens have evolved to subvert or manipulate this mechanism of host defence [19, 20]. Many pathogens master different tricks to either (i) escape the phagosome, such as *Listeria *and *Shigella*, (ii) arrest the phagosomal maturation, either at an early state like *M. tuberculosis *or a late state like *Salmonella *or (iii) manipulate the host cell to transform the phagosome into a customized pathogen-containing vacuole, such as *Legionella, *or *Chlamydia* [19, 20]. 

For* P. acnes* not much is known about the fate it undergoes after engulfment by professional phagocytes. In *in vivo* studies in mice, *P. acnes* could be detected within phagocytic cells at 6 days after injection [21]. Another study found bacteria in the liver and spleen of mice 15 days after intravenous injection [22]. Webster et al. [23] showed that human polymorphonuclear cells (PMNs) and monocytes were unable to degrade *P. acnes in vitro*. In a sarcoidosis study, *P. acnes* was identified intracellularly in alveolar macrophages of the lungs and paracortical macrophages of lymph nodes from sarcoidosis patients [24].

Failure of professional phagocytes to eradicate intracellular *P. acnes*, resulting in bacterial persistence in the host, could lead to chronic tissue inflammation and disease development. In this regard the present study was undertaken to elucidate the intracellular fate of *P. acnes* after ingestion by macrophages and to investigate the failure of professional phagocytes to degrade the bacteria.

## 2. Materials and Methods

### 2.1. Bacterial Culture

For cell culture infection experiments we used* P. acnes* strain P6, isolated from a cancerous prostate [9], strain KPA171202 (DSM 16379) [25], strain 266, a pleuropulmonary isolate [26], and *P. freudenreichii *strain ATCC 6207 (DSM 20271). For mouse prostate inoculations the prostatectomy-derived *P. acnes* strain PA2 was used [10]. Bacteria were cultured on Brucella agar plates (Sigma Aldrich) for three days at 37°C under anaerobic conditions.

### 2.2. Animals

All procedures were performed on 8–10-week-old C57BL/6J wildtype mice under the guidelines of the Johns Hopkins Animal Care and Use Committee (ACUC) and with an approved animal protocol. Mice were housed in a pathogen-free environment, allowed free access to food and water, and were maintained on a 12 h light/dark cycle. Mice were sacrificed via CO_2_ asphyxiation 2 weeks after inoculation of *P. acnes,* and seminal vesicles, urinary bladder, anterior, dorsal/lateral, and ventral prostate lobes were collected along with other reference organs. All tissues were fixed in 10% buffered formalin for 48 h before paraffin embedding.

### 2.3. Transurethral Catheterization and Inoculation of *P. acnes*

Mice were anaesthetized with ketamine/xylazine and then catheterized via the urethra using lubricated sterile polyethylene catheters (PE-10 tubing, BD Biosciences) 2.5 cm in length. Inoculation of *P. acnes* strain PA2 into the bladder and prostate was performed with a dose of approximately 10^7^ colony forming units (CFU) in a 20 *μ*L volume. 

### 2.4. Cell Culture

Cells of the human acute monocytic leukemia cell line THP-1 (DSMZ, ACC 16) were cultured in RPMI medium with L-glutamine and 25 mM 4-(2-hydroxyethyl)-1-piperazineethanesulfonic acid (HEPES) (Gibco Invitrogen GmbH) and supplemented with 10% fetal calf serum (Biochrom AG, Berlin, Germany) and 100 U/mL penicillin/streptomycin (Gibco Invitrogen GmbH) in 75 cm^2^ flasks at 37°C and 5% CO_2_. Phorbol-12-myristate-13-acetate (PMA) (Sigma Aldrich) was added at a concentration of 1 *μ*M to promote differentiation into macrophages at 37°C and 5% CO_2_. After 24 h, PMA was removed by one washing step with culture medium. Fresh medium containing interferon-*γ* (R&B Systems) at a concentration of 150 U/mL was added for another 24 h of incubation to activate macrophages one day before infection. Before *P. acnes* infection, medium containing penicillin/streptomycin was replaced with one washing step with RPMI medium supplemented with 10% FCS without antibiotics.

### 2.5. Intracellular Viability Assay

THP-1 cells were seeded in 6-well plates at 10^6^ cells/well as differentiated and activated cells as described above. Infections were performed at an MOI of 3 at 37°C and 5% CO_2_. Duplicates were performed for each time point and bacterial strain. Infections were stopped, and extracellular bacteria were killed by 3 h incubation with 300 *μ*g/mL gentamicin (Sigma Aldrich) after 24 h. Cells were lysed by a 10 min treatment with 0.5% saponin (Serva Feinbiochemica GmbH and Co. KG). A dilution series was plated on Brucella agar plates (Sigma Aldrich) to determine viable intracellular bacteria by CFU counting after 5 days of incubation at 37°C under anaerobic conditions. Microsoft Excel (Microsoft Corporation, Redmond, USA) and GraphPad Prism 5.0 (GraphPad Software Inc., San Diego, USA) were used to analyse and depict results.

### 2.6. Host Cell Viability

To determine the percentage of dead cells upon *P. acnes* infection over a time course of 3 days, SYTOX green nucleic acid staining (Invitrogen) was applied. Therefore, THP-1 cells were infected with *P. acnes* P6 at an MOI of 3 for 24 h, 48 h and 72 h. Noninfected cells were used as a control. Before staining, cells were washed once with PBS to remove extracellular bacteria. SYTOX green nucleic acid staining was added at a concentration of 2 *μ*M, and emission was measured at a range 485–518 nm with a Fluoroskan Ascent reader (Thermo Labsystems). In the next step all cells were killed by addition of 1% sodium dodecyl sulphate (SDS) to measure the emission of 100% cell death. Percentage of dead cells was then calculated using Microsoft Excel and GraphPad Prism 5.0. 

### 2.7. Detection of Acidified Cellular Compartments with LysoTracker

The acidotropic dye LysoTracker Red DND-99 (Molecular Probes) was used to detect phagosomal acidification. Prior to infection, LysoTracker was diluted in RPMI 1640 medium at 1 : 10000 and added to THP-1 cells for 2 h. THP-1 cells were infected with *P. acnes* P6 with an MOI of 5 for 1 h. Cells were then washed twice with PBS and further incubated in the presence of LysoTracker at 1 : 10000. At 2 h, 6 h, and 24 h postinfection, cells were fixed with 4% paraformaldehyde for 15 min and stained with the anti-*P. acnes* antibody. 

### 2.8. Immunofluorescence (IF)

THP-1 cells were seeded onto sterilized cover slips (Techno Plastics Products AG) with a diameter of 15 mm in a 12-well plate at 5 × 10^5^ cells/well. Cells were differentiated and activated as described above. Bacteria were grown on Brucella agar plates, and infection was performed at an MOI of 3 or 25 at 37°C and 5% CO_2_ under aerobic conditions. Infection was stopped by fixation of cells with 4% paraformaldehyde (PFA) (Sigma Aldrich) in Dulbecco's Phosphate Buffered Saline (PBS) (Gibco Invitrogen GmbH) for 30 min at room temperature (RT). After fixation, PFA was removed by three washing steps with PBS (5 min each), and cells were blocked with 0.3% bovine serum albumin (BSA, Biomol GmbH) in PBS for 1 h at RT. First, extracellular bacteria were stained for 1 h at RT with a 1 : 1000 dilution of a polyclonal mouse anti-*P. acnes* antibody [9]. After three washing steps, extracellular bacteria were labelled with a secondary Cy2-conjugated goat-anti-mouse antibody (Dianova) at a dilution of 1 : 300 for 1 h at RT. Following additional three washing steps, cells were permeabilized for 30 min at RT using 0.1% Triton X-100 (Calbiochem) in PBS containing 0.3% BSA. After permeabilization, intracellular bacteria were stained for 1 h at RT with a 1 : 1000 dilution of the anti-*P. acnes*-antibody followed by three washing steps. Intracellular as well as extracellular bacteria were then labelled by 1 h incubation at RT with a Cy3-conjugated goat-anti-mouse antibody (Dianova). Actin was stained using a 1 : 100 dilution of Alexa Fluor 647 or 568 phalloidin (Invitrogen). Nuclei were stained using a 1 : 1000 dilution of Draq5 (Cell Signaling Technology Inc.) or DAPI. The lysosomal markers LAMP1 and cathepsin D and the early endosomal marker Rab5 were stained with a 1 : 50 dilution of rabbit antibody (Abcam) followed by secondary Alexa Fluor 488 conjugated donkey-anti-rabbit antibody (Invitrogen). After three washing steps, cover slips were mounted in Mowiol and stored at 4°C. Immunofluorescence staining was analyzed by confocal laser scanning microscopy using a Leica TCS SP equipped with an argon-krypton mixed gas laser. 

### 2.9. Immunohistochemistry (IHC)

Slides containing sections of formalin-fixed, paraffin-embedded (FFPE) mouse prostate tissues were deparaffinized in xylene and rehydrated through a series of graded ethanol. For F4/80 and *P. acnes *IHC, slides were steamed for 40 min (anti-F4/80 antibody) or 17 min (anti-*P. acnes* antibody [9]) in high temperature target retrieval solution for antigen retrieval (Dako Cytomation). Slides were then incubated with primary antibodies against F4/80 (AbD Serotec) at a dilution of 1 : 10,000 for 45 min at room temperature or against *P. acnes *at a dilution of 1 : 2000 overnight at 4°C. For F4/80 IHC, slides were incubated with a rabbit polyclonal linker antibody to Rat IgG (Abcam) at 1 : 400. Slides were then incubated with secondary antibody (PowerVision, Leica Microsystems) for 45 min at room temperature. Staining was visualized using 3,3′-Diaminobenzidine (Sigma FAST 3,3′-Diaminobenzidine tablets), and slides were counterstained with hematoxylin.

## 3. Results

### 3.1. Detection of* P. acnes* in Macrophages *In Vivo*

**Figure 1:**
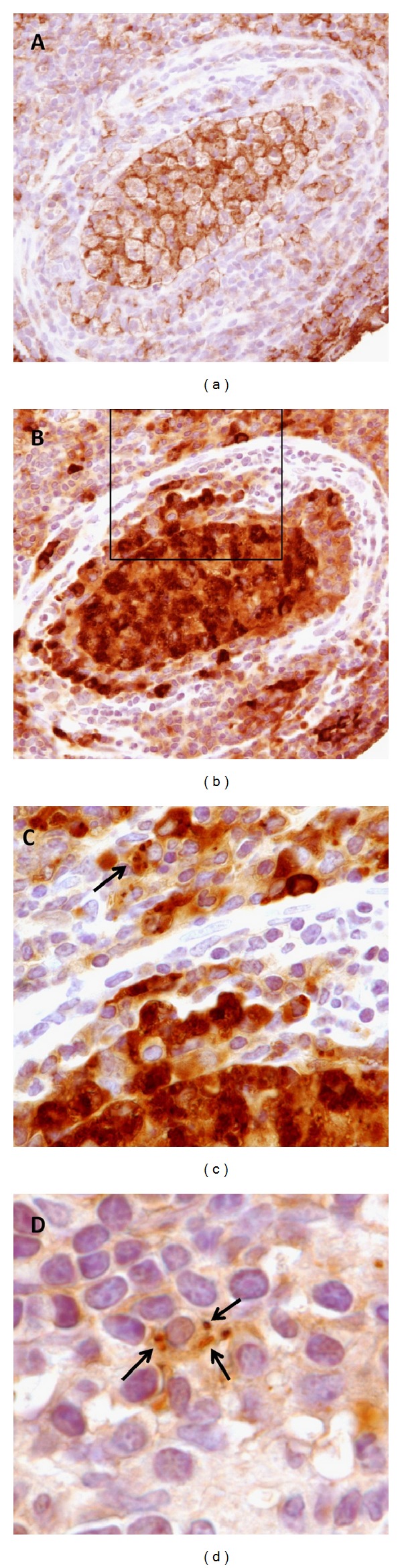
Presence of *P. acnes* in mouse prostate-infiltrating macrophages. (a) Inflamed dorsal prostate gland 2 weeks after infection with *P. acnes*. Glandular lumen contains primarily macrophages as indicated by IHC for F4/80 macrophage marker. (b) Adjacent section stained with an anti-*P. acnes* antibody. Note dense accumulation of *P. acnes* cells in macrophages within glandular lumen. (c) Enlarged view of boxed area in (b). Single *P. acnes* cells can be visualized within prostate-infiltrating macrophages (arrow). (d) Additional image of *P. acnes* cells (arrows) in prostate-infiltrating macrophage as indicated by IHC with anti-*P. acnes* antibody.

Recently, a mouse model of chronic prostatic inflammation was established [27], using the prostate-derived *P. acnes* isolate PA2 as infectious agent. At 2 weeks postinfection (p.i.), *P. acnes* could be detected in prostate-infiltrating macrophages as assessed by IHC with an anti-*P. acnes* antibody (Figure 1). Infiltrating macrophages in the glandular lumen contained multiple *P. acnes* cells (Figures 1(c) and 1(d)). 

### 3.2. A Clinical Prostate Isolate of *P. acnes* Persists Intracellularly in the Human Macrophage Cell Line THP-1

**Figure 2:**
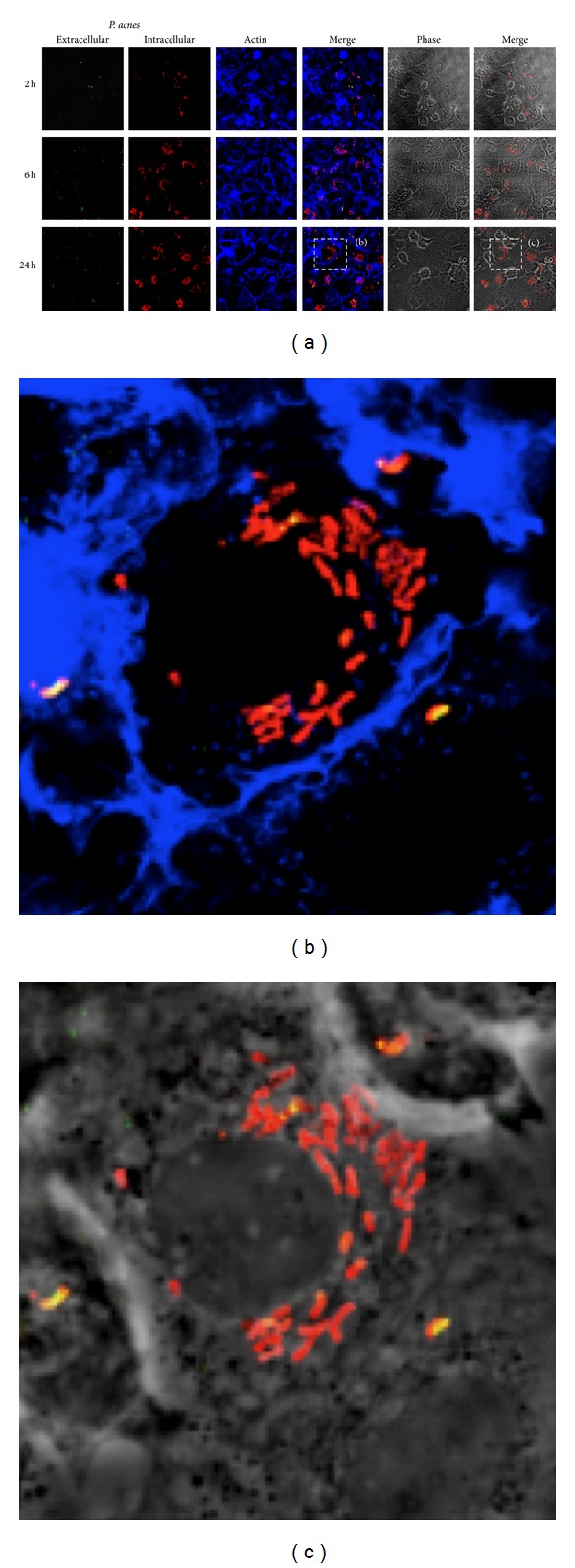
Infection of THP-1 cells with prostate isolate *P. acnes* P6. (a) THP-1 cells were infected with *P. acnes* P6 at an MOI of 25. Infection was stopped after 2, 6, and 24 h. Cells were fixed with 4% PFA, and extracellular bacteria were stained with mouse polyclonal anti-*P. acnes* antibody and Cy-2 labelled goat-anti-mouse antibody (green). After permeabilization, intracellular bacteria were stained with mouse polyclonal anti-*P. acnes* antibody and Cy-3 labelled goat-anti-mouse antibody (red). Overlay of both stainings indicates extracellular bacteria (appearing yellow). Actin was stained with Alexa Fluor 647 phalloidin (blue). (b) and (c) Zoom-in on intracellular *P. acnes* (red) at 24 h p.i.

A macrophage cell culture model was established to investigate the intracellular fate of *P. acnes*. We first wanted to visualize the uptake and persistence of the clinical *P. acnes* strain P6 in the human macrophage cell line THP-1. The cells were differentiated by PMA treatment and stimulation with interferon gamma for 24 h prior to infection. Intracellular and extracellular bacteria could be detected by immunofluorescence (IF) staining at p.i. time points of 2 h, 6 h, and 24 h (Figure 2). Intracellular numbers of viable *P. acnes* increased between 2 h and 6 h p.i. as judged from colony forming unit (CFU) counts (data not shown). Massive intracellular clusters were present at 6 h and 24 h p.i. and were located around the nucleus (Figures 2(b) and 2(c)). 

### 3.3. Absence of Intracellular Replication and Escape from the Host Cell

To investigate the possibility of intracellular replication or bacterial escape from the host cell, THP-1 cells were infected with *P. acnes* for 1 h, and extracellular bacteria were killed with antibiotic treatment for 2 h. The culture medium was replaced with antibiotic free medium, and the number of intracellular bacteria and escaped bacteria in the medium were determined after 2 h, 6 h, and 24 h of infection by CFU counts. We did not observe intracellular replication of *P. acnes* nor bacterial escape from the host cell (data not shown).

### 3.4. Comparison of Intracellular Viability of Different *P. acnes* Wild-Type Strains and *P. freudenreichii*

**Figure 3:**
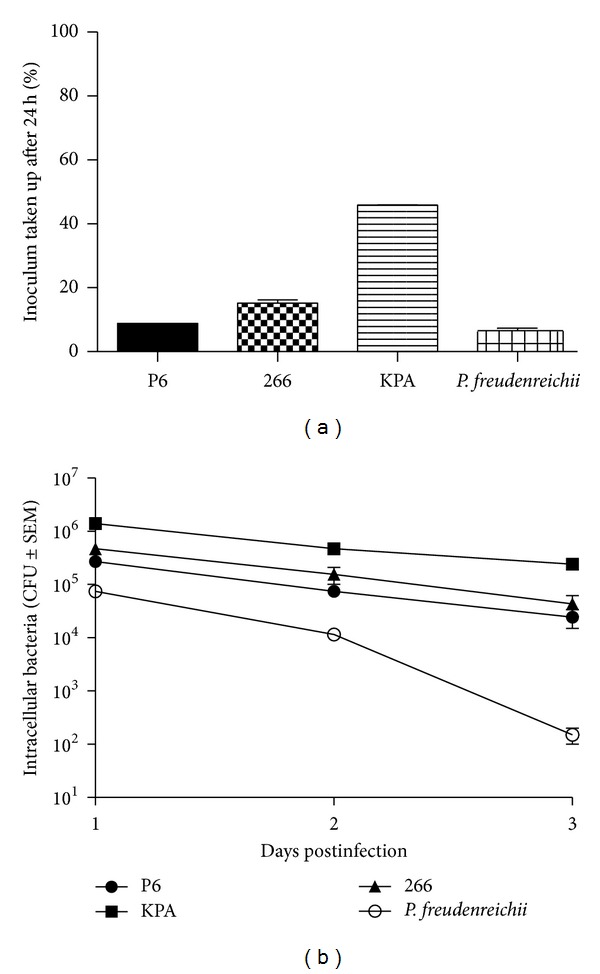
Intracellular viability of propionibacteria in THP-1 cells. THP-1 cells were infected with *P. acnes* strains P6, KPA and 266, and *P. freudenreichii* at an MOI of 3. Extracellular bacteria were killed after 24 h by 3 h incubation with 300 *μ*g/mL gentamicin. 100 *μ*g/mL gentamicin was kept in the medium at all times to prevent reinfection with extracellular bacteria. Cells were lysed with 0.5% saponin, and remains were scraped off and mixed by vortexing. A dilution series was plated out on Brucella agar plates to determine viable intracellular bacteria by determining CFUs after 5 days of incubation at 37°C under anaerobic conditions. Data were evaluated using Microsoft Excel and GraphPad Prism. Values represent mean of *n* = 2 ± SEM. (a) Presence of intracellular bacteria at 24 h p.i., compared to original inoculum (100%). (b) CFU counts of intracellular bacteria over a time course of 3 days.

To investigate if intracellular persistence in macrophages is a trait common to *P. acnes* strains, we chose three strains, isolated from distinct body sites and belonging to different multilocus sequence types (MLSTs): the clinical prostate isolate P6 (ST33, type I-2), the skin isolate KPA171202 (ST34, type I-2), and the lung isolate 266 (ST18, type I-1a) [9, 25, 26]. In addition, a comparison was made to the dairy *Propionibacterium freudenreichii *(*P. freudenreichii*), which is not associated with humans, in order to assess if persistence is a trait attributed to all *Propionibacteria* or to *P. acnes* alone. We also wanted to test if intracellular bacteria are still alive and able to replicate after isolation from macrophages, which could be important for the persistence in host tissue and potentially for invasion of other cell types. After 24 h p.i., extracellular bacteria were killed by antibiotic treatment, and presence of live intracellular bacteria was determined over a time course of 3 days by plating of cell lysates on Brucella agar plates and CFU counts. *Propionibacteria *of all 4 strains recovered from THP-1 cells were viable and able to replicate on agar plates. The initial inoculum of each strain was calculated and set to 100%. At 24 h p.i. viable bacteria could be found intracellularly: 8.9% (±0.1%) of the initial inoculation of strain P6, 45.9% (±0.1%) of KPA, 15.2% (±1.4%) of 266, and 6.3% (±1.2%) of *P. freudenreichii*, showing strain-dependent differences in the uptake rate by macrophages (Figure 3(a)). No increase in CFU was observed for any of the used strains over the time course, indicative of the absence of intracellular replication. The CFU of intracellular *P. acnes* stayed stable over 3-day time course, while the CFU of *P. freudenreichii* dropped more than 2 logs from day one to day 3 (Figure 3(b)). This experiment showed that intracellular survival and persistence is common to *P. acnes* strains but not to *P. freudenreichii.*

### 3.5. Persistence of *P. acnes* in THP-1 Cells Does Not Cause Host Cell Death

In order to observe host cell fitness and survival during long-term *P. acnes* infection, a SYTOX green nucleic acid staining was performed on *P. acnes* strain P6-infected THP-1 cells at 24 h, 48 h, and 72 h p.i. At 24 h p.i., 6.1% (±0.1%) of dead cells were observed in P6-infected THP-1 cells, compared to 4.7% (±0.2%) in noninfected cells (data not shown). This number increased to 7.0% (±0.9%) in infected and 6.5% (±0.3%) in noninfected cells at 72 h p.i. Thus, *P. acnes* P6 infection does not damage or kill THP-1 cells.

### 3.6. Transient Acidification of* P. acnes*-Containing Phagosomes

**Figure 4:**
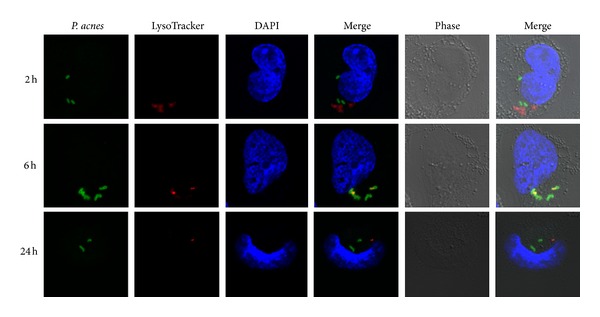
Confocal analysis of LysoTracker-treated THP-1 cells infected with* P. acnes.* To visualize acidified cellular compartments the acidotropic dye LysoTracker Red DND-99 was used (red). Intracellular *P. acnes* were stained in green. Colocalization of *P. acnes* and LysoTracker, indicative of an acidified *P. acnes*-containing phagosome, was apparent at 6 h p.i. (yellow/orange) but not at 2 h and 24 h p.i. Shown are representative images from two independent infection experiments.

The observed persistence of *P. acnes* in activated THP-1 cells indicates that *P. acnes* is able to prevent or delay intracellular degradation. Upon engulfment by THP-1, bacteria are typically located in phagosomes. Via recurrent fusion and fission events with compartments of the endocytic pathway, the bacteria-containing phagosomes get gradually acidified in order to arrest the growth of entrapped bacteria and activate degrading enzymes for bacterial lysis [16–20]. To assess the process and state of acidification of the *P. acnes*-containing phagosomes, we performed infections of THP-1 cells in the presence of the acidotropic dye LysoTracker Red DND-99 that stains acidified cellular compartments. At early time points (2 h p.i.) no acidification of *P. acnes*-containing compartments can be detected, but at 6 h p.i. most bacteria-containing phagosomes are acidified (Figure 4). Interestingly, the acidification is gone at later time points (24 h p.i.), which could indicate the bacterial escape from phagosomes or intraphagosomal pH neutralization.

### 3.7. Intracellular *P. acnes* Do Not Colocalize with the Lysosomal Marker LAMP1 in THP-1 Cells 24 h p.i

**Figure 5:**
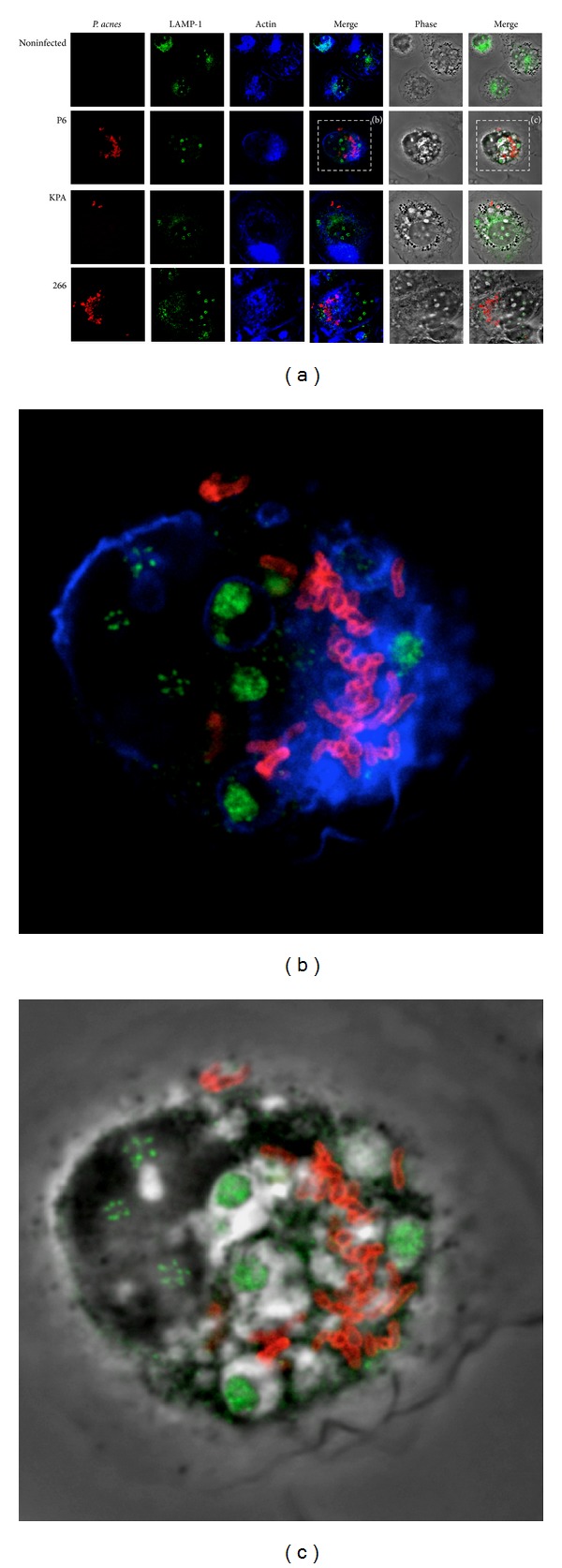
Confocal analysis of *P. acnes* and LAMP1 in THP-1 cells at 24 h p.i. (a) THP-1 cells were infected with *P. acnes* wild-type strains P6, KPA and 266 at an MOI of 3 for 24 h. Extracellular bacteria were killed with 300 *μ*g/mL gentamycin and removed by washing steps. Cells were fixed, permeabilized, and intracellular bacteria were stained with mouse polyclonal anti-*P. acnes* antibody and Cy-3 labelled donkey-anti-mouse antibody (red). The lysosomal marker LAMP1 was stained with rabbit-anti-LAMP1 antibody and Alexa Fluor 488 conjugated donkey-anti-rabbit-antibody (green). Actin was stained with Alexa Fluor 647 phalloidin (blue). (b) and (c) Zoom-in on cells infected with *P. acnes* P6.

In order to further characterize the state of intracellular *P. acnes* at the time of 24 h postinfection we investigated the presence of a typical markers of late phagosomes, the lysosome-associated membrane protein 1 (LAMP1). This protein is one of the most abundant integral membrane proteins of phagolysosomes and has been shown to be essential for successful maturation from early to late stages [28]. Immunofluorescence staining of intracellular bacteria and LAMP1 was performed at 24 h p.i. No colocalization with LAMP1 was observed (Figure 5). This result supports the hypothesis that intracellular *P. acnes* do not reach the state of typical phagolysosomes.

### 3.8. Intracellular *P. acnes* in THP-1 Cells Do Not Colocalize with the Lysosomal Marker Cathepsin D at 24 h p.i

**Figure 6:**
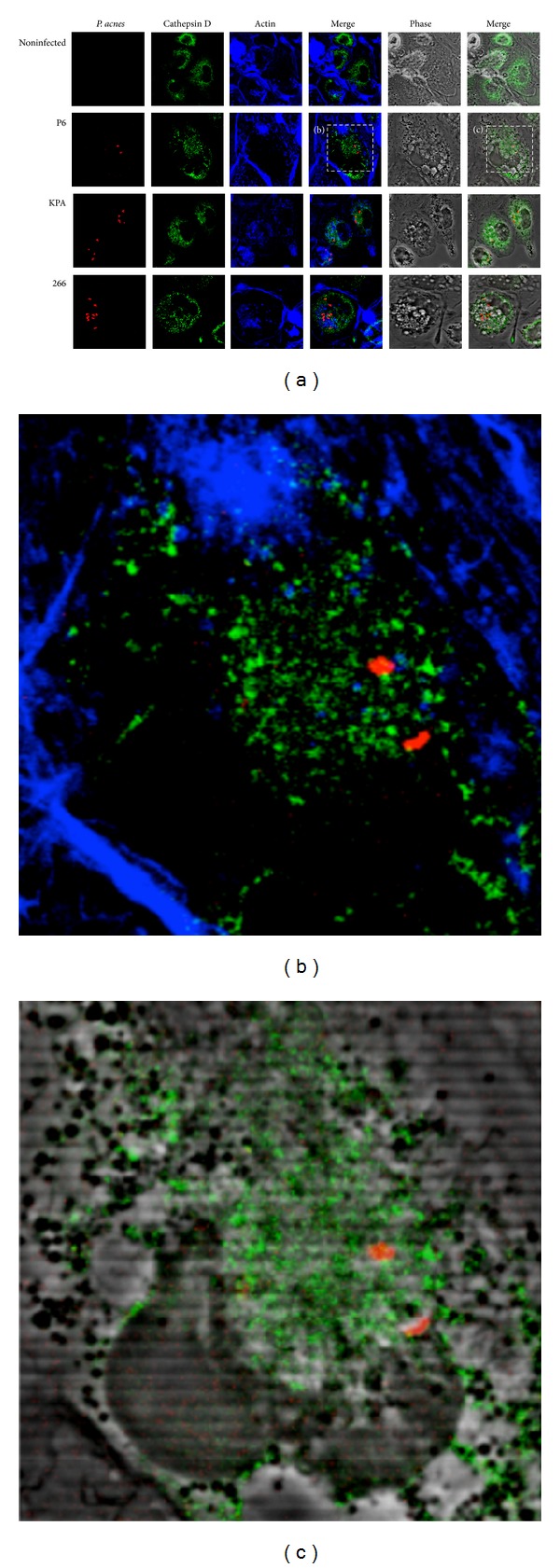
Confocal analysis of *P. acnes* and cathepsin D in THP-1 cells at 24 h p.i. (a) Experiments were performed as described in Figure 5 legend. The lysosomal marker cathepsin D was stained with rabbit-anti-cathepsin D antibody and Cy2-conjugated donkey-anti-rabbit antibody (green). Intracellular *P. acnes* P6 (red), actin (blue). (b) and (c) Zoom-in on cells infected with *P. acnes* P6.

To further strengthen the hypothesis that intracellular *P. acnes *do not end up in functional phagolysosomes we performed IF staining of the aspartic endopeptidase cathepsin D, which is a typical hydrolytic enzyme found in mature phagolysosomes [29]. Again, no colocalization of cathepsin D with intracellular *P. acnes* was observed (Figure 6). 

### 3.9. Intracellular *P. acnes* in THP-1 Cells Do Not Colocalize with the Early Endosomal Marker Rab5 at 24 h p.i

**Figure 7:**
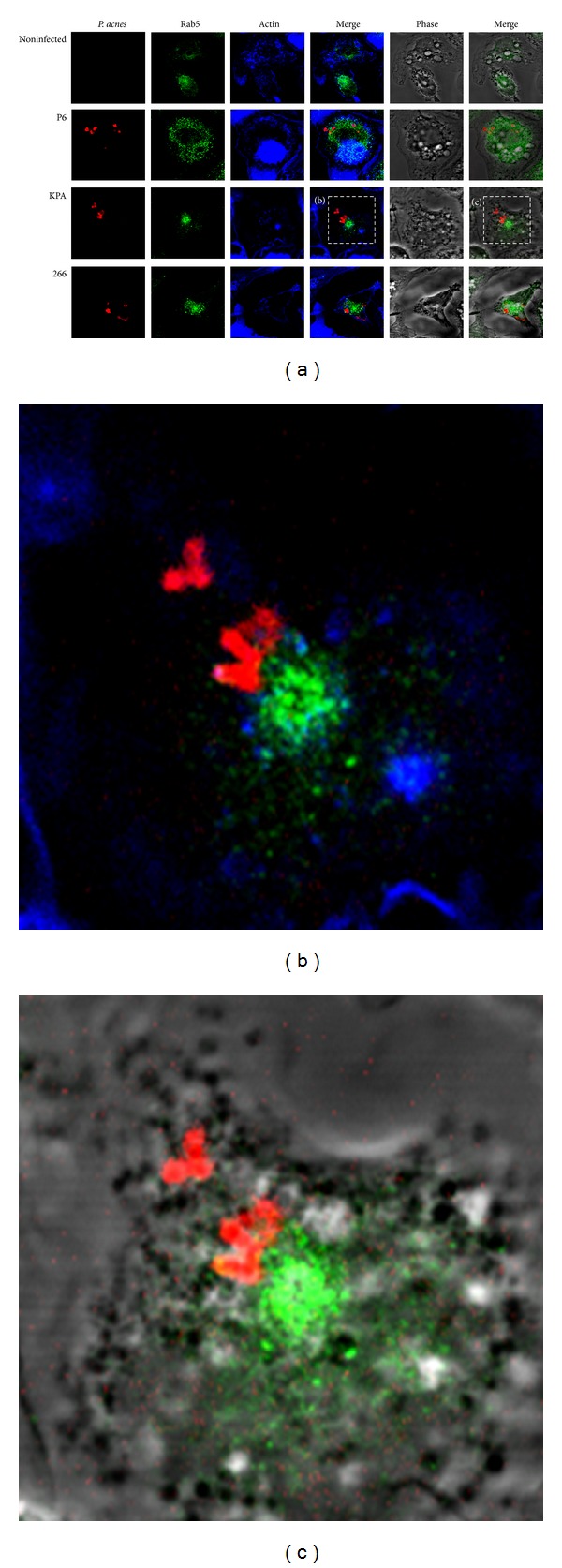
Confocal analysis of *P. acnes* and Rab5 in THP-1 cells at 24 h p.i. (a) Experiments were performed as described in Figure 5 legend. The early endosome marker Rab5 was stained with rabbit-anti-Rab5 antibody and Alexa Fluor 488 conjugated donkey-anti-rabbit antibody (green). Intracellular *P. acnes* P6 (red), Actin (blue). (b) and (c) Zoom-in on cells infected with *P. acnes* P6.

We did not find any colocalization of *P. acnes* with prominent lysosomal markers. Thus, we decided to investigate the possibility of a phagosomal maturation arrest in an early stage. This was shown for the persistent intracellular pathogen *Mycobacterium tuberculosis*; bacteria-containing vacuoles acquire the early endosome marker Rab5 but no markers of late endosomes or lysosomes [30]. IF staining of Rab5 and intracellular *P. acnes* in THP-1 cells at 24 h p.i. showed no colocalization (Figure 7). This suggests that *P. acnes* persistence and survival in macrophages is not due to an arrest at an early phagosomal stage. We also wanted to investigate the possibility of early acquisition and rapid loss of Rab5, as described for some other pathogens including *Salmonella* species. Synchronized phagocytosis was stopped after 15, 30, and 45 min and 1 and 2 h. The staining pattern of Rab5 did not change over the time course observed (data not shown). However, confocal analysis showed only very few intracellular *P. acnes* at the respective times; thus we cannot rule out the possibility of early Rab5 acquisition and subsequent rapid loss. 

## 4. Discussion 

The intracellular lifecycle of *P. acnes* has not been studied in much detail so far. Fassi Fehri et al. [9] performed electron microscopy of the prostate epithelial cell line RWPE1, where *P. acnes* was located in intracellular vacuoles, either as single bacteria or in small clusters. These findings were similar to results obtained by Tanabe et al. [14], who showed that *P. acnes* isolates from sarcoidosis patients can invade the epithelial cell lines A549 and HRK293T. Previous research has also shown that professional phagocytes fail to clear *P. acnes*; not only are PMNs and monocytes unable to degrade *P. acnes in vitro* [23], but also intact bacteria have been found intracellularly in murine macrophages 6 days after injection [21]. A recent study showed that *P. acnes* can lead to a chronic inflammation of the mouse prostate; introduction of *P. acnes* into the mouse prostate via transurethral catheterization and inoculation led to chronic inflammation of the dorsal lobe of the prostate that persisted for at least 8 weeks after inoculation [27]. Similar results have been obtained in a rat model [31]. These observations provide evidence for the existence of *P. acnes* mechanisms to promote intracellular survival and persistence.

We showed here that *P. acnes* can be detected *in vivo* in prostate-infiltrating macrophages of the mouse at 2 weeks after *P. acnes* inoculation. In the human macrophage cell line THP-1 *P. acnes* not only survives but also persists. There are no signs of intracellular replication or escape from the host cell, and the presence of intracellular bacteria does not cause host cell death. Regarding the fate of THP-1 engulfed *P. acnes,* we detected intracellular acidification of *P. acnes*-containing compartments, which was transient. We could not detect colocalization with the late lysosomal markers LAMP1 and cathepsin D, or with the early endosomal marker Rab5 after 24 h of infection. The nonpathogenic relative *P. freudenreichii* is slowly degraded by THP-1 cells, which hints at a *P. acnes*-specific trait that confers protection to phagosomal degradation. Our data suggest a *P. acnes*-specific diversion or blockage of the phagosome maturation pathway and a possible bacterial escape from the phagosomal compartment. We could not reveal in this study at which step the maturation of *P. acnes*-containing phagosomes stops or diverges from the typical maturation pathway. 

Intracellular bacteria persist but do not multiply within or escape from the host cell or cause host cell death. These findings underline the assumption that *P. acnes* is not a typical intracellular pathogen but still confers means to avoid the phagolysosomal degradation machinery. Identifying differences in uptake and persistence of different strains/phylotypes of *P. acnes* could help to elucidate the mechanism of protection from degradation. The comparison of *P. acnes* strains indicated strain-specific uptake efficiencies. But the mechanism to evade degradation seems to be conserved and present in all three wild-type *P. acnes* strains. On the other hand, *P. freudenreichii* does not possess the *P. acnes* survival trait and was slowly degraded in THP-1 cells. The genome of *P. freudenreichii* has been sequenced [32]; 60% of *P. acnes* coding sequences have an ortholog in *P. freudenreichii*. However, none of the putative virulence factors of *P. acnes* such as sialidases, dermatan-sulphate adhesins, and Christie-Atkins-Munch-Perterson (CAMP) factors were identified in *P. freudenreichii*. We hypothesise that the *P. acnes*-specific survival trait is mediated either by a *P. acnes-*specific virulence factor or by differences of the cell wall composition. It was proposed in the 1980s that intracellular persistence of *P. acnes* is a result of the thick and tightly cross-linked cell wall that is resistant to the action of degrading and oxidizing enzymes of the host defence machinery [23, 33]. 

We observe acidification of the *P. acnes*-containing compartment at 6 h postinfection; at later time points (24 h p.i.) this was reverted as we detected no signs of acidification surrounding the intracellular location of *P. acnes*. One explanation of this observation is a *P. acnes* mechanism to counteract intraphagosomal acidification. Interestingly, a previous study identified a *P. acnes* system that is highly expressed and that can neutralize acidic conditions: the arginine deiminase (ADI) pathway [26]. This system converts arginine to ornithine, thereby generating NH_3_ and ATP. The ADI pathway of *Listeria monocytogenes* has been implicated in the bacterial survival within macrophage phagosomes [34]. Another possibility is a bacterial escape from the phagosome. Interestingly, a recent study by Nakatsuji et al. suggests that CAMP factors of *P. acnes* could act together with a membrane-associated form of mammalian sphingomyelinases and facilitate phagosomal escape of *P. acnes* [35]. CAMP factors possess cohemolytic activity and have been reported to be pore-forming toxins [36, 37].

## 5. Conclusions 

The present study allows a first insight into possible check points of interference or blockage of the phagosomal maturation pathway in macrophages by *P. acnes* and opens up many interesting questions about the *P. acnes *intracellular lifecycle, including the intriguing possibility that *P. acnes* uses macrophages as a niche for survival and spread. Further investigations will help us to identify and understand the underlying mechanisms of intracellular survival and persistence and might help to develop strategies of *P. acnes* eradication in diseased patients. This could bring helpful innovations in the fight against chronic *P. acnes* infections and the associated pathologies.
